# How to capture developmental brain dynamics: gaps and solutions

**DOI:** 10.1038/s41539-021-00088-6

**Published:** 2021-05-03

**Authors:** Nienke van Atteveldt, Maaike Vandermosten, Wouter Weeda, Milene Bonte

**Affiliations:** 1 Dept. of Clinical Developmental Psychology & Institute Learn!, Vrije Universiteit Amsterdam, Amsterdam, The Netherlands; 2 Dept. of Neuroscience, and Leuven Brain Institute, Experimental ORL, KU Leuven, Leuven, Belgium; 3 Dept. of Methodology & Statistics, Leiden University, Leiden, The Netherlands; 4 Dept. of Cognitive Neuroscience, and Maastricht Brain Imaging Center, Faculty of Psychology and Neuroscience, Maastricht University, Maastricht, The Netherlands

**Keywords:** Development of the nervous system, Human behaviour, Cognitive neuroscience, Conferences and meetings

## Abstract

Capturing developmental and learning-induced brain dynamics is extremely challenging as changes occur interactively across multiple levels and emerging functions. Different levels include the (social) environment, cognitive and behavioral levels, structural and functional brain changes, and genetics, while functions include domains such as math, reading, and executive function. Here, we report the insights that emerged from the workshop “Capturing Developmental Brain Dynamics”, organized to bring together multidisciplinary approaches to integrate data on development and learning across different levels, functions, and time points. During the workshop, current main gaps in our knowledge and tools were identified including the need for: (1) common frameworks, (2) longitudinal, large-scale, multisite studies using representative participant samples, (3) understanding interindividual variability, (4) explicit distinction of understanding versus predicting, and (5) reproducible research. After illustrating interactions across levels and functions during development, we discuss the identified gaps and provide solutions to advance the capturing of developmental brain dynamics.

## Introduction

**Fig. 1:**
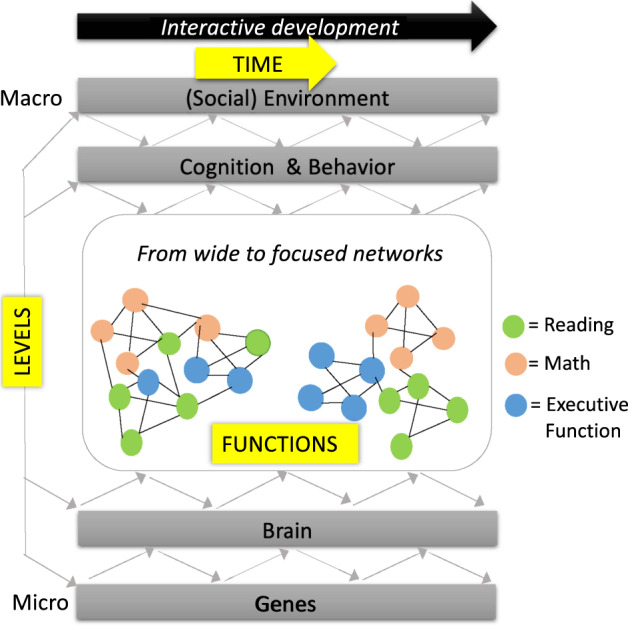
Example framework that unifies developmental changes across levels and functions. During development (TIME dimension), there is continuous interaction across levels of change (LEVELS: gray bars) as well as across emerging functions (FUNCTIONS: presented as interactive networks). Reading, math, and executive function are chosen as examples of interactively emerging functions during development. Thus, during development, the neural and cognitive correlates of these different functions are first characterized as wide, overlapping networks which then gradually specialize to more focused networks with learning and maturation (see section “Bidirectional interaction across functional networks”).

Understanding how children develop and learn is of tremendous importance not only for preventing and remediating disorders, but also to inform education practices and parenting guidelines. However, capturing developmental and learning-induced dynamics is extremely challenging. In April 2019, theoretical and methodological advances to address this challenge and remaining gaps and solutions were explored in the workshop “Capturing Developmental Brain Dynamics” at the NIAS-Lorentz center in Leiden, the Netherlands. The workshop consisted of an alternation between keynote presentations to provide the state-of-the art in different topics (see Table 1), and interactive elements (such as an Open Space event, working group break-out sessions), in which participants from different disciplines interacted to define gaps and solutions. One defining characteristic of brain development that recurred throughout the entire workshop, is the enormous extent of continuous interactions not only occurring across levels (e.g., across genetic and brain levels^1^), but also across the different emerging functions (e.g., language, reading, math, executive function^2^). In Fig. 1, we illustrate these two dimensions of interactivity: (1) interacting levels in the entire range from micro- to macro: genes, brain function and structure, behavior, cognition and, (social) environment and (2) interacting functions, where the neural and cognitive correlates of each function are first characterized by wide networks with overlapping nodes between the functions and showing a gradual specialization to more focused networks over time. In Table 1, we provide an overview of how the work of the different workshop presenters relates to the different levels and functions of Fig. 1.

**Table 1:** An overview of speakers during the workshop and how their work relates to the different levels and functions in Fig. 1.

Speaker	Topic	Level(s) of research (Fig. 1)	Function(s) (Fig. 1)	References in this report
Takao Hensch, Harvard University	Micro-biological changes	Brain (molecular), Genes, Environment	Visual and auditory perception	Morishita et al., 2010; Werker & Hensch 2015
Mark Johnson, University of Cambridge	Macro-biological changes	Brain (anatomical-functional)	Face/voice perception, social cognition, autism	Johnson, 2011; Johnson et al., 2015
Nadine Gaab, Harvard University	Language development and dyslexia	Brain (functional), Environment	Language, reading, dyslexia	Ozernov-Palchik et al., 2016
Silvia Brem, University of Zurich	Visual cortex changes by print exposure & reading	Brain (functional)	Reading	Brem et al., 2010; Chyl et al. 2021 Maurer et al., 2007; Pleisch et al., 2019.
Bert De Smedt, University of Leuven	Development of math cognition and dyscalculia	Brain (functional), Cognition, Behavior	Math (dyscalculia)	Ashkenazi et al., 2017; Peters et al., 2018; Peters et al., 2020.
Iro Xenidou-Dervou, Loughborough University	Structural equation modeling and growth models	Cognition, Behavior	Methods focus (math, working memory)	Xenidou-Dervou et al., 2018.
Chris van Klaveren, Vrije Universiteit Amsterdam	Predictive modeling and machine learning	Cognition, Behavior	Methods focus	Cornelisz et al., 2020.
Niko Steinbeis, University College London	Development of self-control	Brain (anatomical, functional), Behavior	Cognitive control, decision-making	Smid et al., 2020.
Barbara Braams, Vrije Universiteit Amsterdam	Risk-taking during adolescence and real-life neuroscience	Brain (functional), Behavior, Environment	Risk-taking, social cognition	Braams et al. 2019; van Atteveldt et al., 2018.
Dirk Smeets, Icometrics	Longitudinal structural MRI	Brain (anatomical)	Methods focus	Phan et al., 2018.
Kate Mills, University of Oregon	Longitudinal functional MRI	Brain (anatomical, functional), Behavior	Methods focus (risk-taking)	Klapwijk et al., 2021.
Rogier Kievit, Cambridge University	Brain-behavior interactions during development	Brain (anatomical), Cognition, Behavior	Methods focus (cognitive ability)	Kievit et al., 2017; 2018; 2019; 2020.
Michael Skeide, Max Planck Institute	Integrating genetic and neuroimaging data	Genes, Brain (anatomical)	Methods focus (math, reading)	Skeide et al., 2020.
Tom Wilderjans, Leiden University	Clustering multi-subject brain data with ICA	Brain (functional)	Methods focus	Durieux & Wilderjans, 2019.

The importance of interactivity during human development has been described by many others before. For example, interactions across levels have been described in integrated theories of development, such as Sameroff’s unified theory of development^3^, Bronfenbrenner’s ecological systems theory^4^, or Gottlieb’s multilevel transactional model^5^. Dynamic interactions between cognitive functions during development have also been described, more generally (e.g., interactive specialization^6^) or in the context of specific cognitive functions (e.g., mutualism^7^) or neurodevelopmental disorders^8,9^. However, as identified throughout the workshop, there is a clear need for a unifying framework that fits all of development (rather than one or two specific levels or functions/disorders) and considers both dimensions of interactive change (levels and functions) as well as the developmental dimension (time), as illustrated in Fig. 1. It was exciting to see the progress that has been made with respect to the availability of analytical tools needed to quantitatively test interactive developmental changes across multidimensional datasets in large numbers of children. At the same time, we realized that combining tools and knowledge from different disciplines is still challenging. This report aims to stimulate such endeavors also beyond the workshop participants. Below, we first illustrate the two dimensions of bidirectional interaction (levels, functions) during development with several examples, inspired by the workshop presenters and discussions. Next, we discuss the gaps identified during the workshop, and solutions to advance the capturing of developmental brain dynamics.

### Bidirectional interaction across levels

One of the challenges in understanding development is the complexity and malleability resulting from interactive changes across levels (Fig. 1, gray bars). Thus, instead of a deterministic view in which there is a unidirectional causal path from genes to brains to behavior, development can only be understood when considering bidirectional interactions between each of these levels, called circular causality^10^. Even a classical “biologically driven” developmental stage such as a critical period, turns out to be malleable due to genetically induced plasticity and environmental influences^11^. An example of a genetically induced shift in plasticity is provided by knock-out mice in which deletion of a protein that normally acts as a brake on visual cortical plasticity resulted in animals that displayed recovery from amblyopia (lazy eye) at an age far beyond the typical critical period for such plasticity^12^. A striking example of experientially induced plasticity is given by unexpected improvements in stereoacuity in 10-year-old amblyopic children as a result of 20 h of playing fast-paced and highly engaging action video games^13^.

Understanding the timing and mechanisms of genetically and experientially induced change in learning capacity may inform optimal timing of interventions. For example, as discussed by Ozernov-Palcik et al.^14^, reading outcomes in children with developmental dyslexia may be substantially improved if we manage to anticipate difficulties through early screening and provide treatment during the most effective intervention window, i.e., at the very start of reading acquisition rather than after years of reading failure, which is current clinical practice. Although individual differences in the progression of structural-functional brain development may lead to variability in the precise timing of this window^15^, intervention-induced reading gains tend to be optimal for intervention between kindergarten and grade 2, after which these gains tend to stabilize^16–18^.

During the workshop, bidirectional and dynamic interaction across levels was also addressed by Johnson^6^, who argued that a purely maturational account of development, which assumes a unidirectional pathway from anatomical development to the emergence of functions, is too simplistic and static. In line with the dynamic and interactive nature of development, increasing evidence shows that often it is not that a child can’t use a certain function or behavior yet (such as a strategy), but more that it won’t use it, unless in specific circumstances. These circumstances are often influenced by the environmental level, and in the case of adolescents, the social environment of peers. For example, while brain maturation may result in a certain propensity for risk-taking behaviors in adolescence, the social environment (e.g., choices of peers) may define their actual risk-taking behaviors^19,20^. Another example discussed during the workshop is provided by developmental studies into decision-making strategies. These studies typically compare the use of model-free (habitual, rigid but cheap) and model-based (goal-directed, flexible but costly) learning at different ages. Several studies found that model-based learning does not emerge until adolescence^21,22^, yet when model-based learning is tested in a simplified paradigm and with higher rewards, children as young as 5 years used these strategies^23^. These examples emphasize the importance of connecting developmental research to a child’s real-life situation, i.e., to include the environment level^24^.

Finally, the view of circular causality across levels can also help explaining neurodevelopmental disabilities. For example, in autism widespread brain dysfunction affects how social stimuli are processed and this in its turn influences which input children with autism select. Given that a child will seek for aspects of the environment that (s)he is able to predict, a child with autism is biased towards learnable environments that generally have simpler structures and that include repetitive behaviors, at the cost of actively selecting situations that include more complex social interactions^25^.

### Bidirectional interaction across functional networks

In addition to interactions between the different levels as illustrated above, the interactions between different functional networks also play a crucial role during development. At the brain level, the tight functional interaction between different cortical regions during development, results in increasingly specialized regions that display increased responses to certain stimuli or tasks but decreased responses to non-preferred stimuli or tasks (i.e., from wide to focused networks in Fig. 1; see also^6^). Evidence for this framework is provided for example in the domain of literacy acquisition. Dehaene-Lambertz et al.^26^ collected longitudinal fMRI data in ten young children prior, during, and after the first year of learning to read. Simultaneous with the acquisition of reading skills, a specialization for words started to emerge in the visual word form area (VWFA). Prior to reading, this region was weakly specialized for tools, but once reading acquisition started, development of the initial function was inhibited, while a specialization for reading emerged. During the workshop it was discussed how the exact location of this specialized VWFA is determined by its connectivity to other brain regions involved in spoken language functions^27^, hence the close interaction between reading and spoken language functions is crucial to form a specialized network. The specialization for print was further shown to develop rapidly through association training, to depend on learning performance^28–31^, and to be reduced in children with dyslexia^30–32^. Together, these findings suggest that interactive specialization seems to be a crucial step for adequate reading development.

At the behavioral level, interactive development across functions is supported by the findings of mutualism between cognitive domains^33,34^. Mutualism explains how improvements in different cognitive functions, such as vocabulary and reasoning, benefit each other over time. Another example of how functions interactively develop is comorbidity. Traditionally, classification systems such as the Diagnostic and Statistical Manual of Mental Disorders (e.g., DSM IV^35^) assigned a disorder to an individual if they exhibit a number of specific symptoms, and comorbidity is identified if the symptoms of another disorder are also met. Although the same logic of listing specific diagnostic criteria still holds, this strict division is less present in the most recent DSM-V edition^36^, which for example groups both dyslexia and dyscalculia under the same category of specific learning disabilities. Alternative diagnostic frameworks based on recent advances take a different approach by looking at groups of symptoms as part of an interactive network spanning multiple disorders. Then, the relationship between symptoms is what constitutes a disorder, and comorbidity is automatically incorporated since common symptoms are now part of the same network. This approach enables better identification of relevant targets for therapeutic intervention. For example, network analysis of depression and anxiety disorder, two highly comorbid disorders, showed that lack of sleep was one of the most central symptoms in the depression/anxiety network^37,38^. Improving sleep therefore potentially improves other symptoms in both disorders.

In the workshop this shift from a classical categorical approach towards a continuous model of dysfunction was specifically discussed with respect to neurodevelopmental disorders such as dyslexia and dyscalculia, that affect overlapping domain general cognitive skills as well as more specific skills such as phonological and magnitude processing that may differentiate with reading and math development (i.e., wide to focused networks in Fig. 1; see^39–41^. Thus, according to such a continuous model we need to consider individual differences across the entire spectrum, with disorders such as dyslexia and dyscalculia representing respective lower ends of a continuum, rather than a qualitatively different condition^42,43^. Whether, and to what extent, a child develops difficulties then depends on a combination of biological, cognitive, and environmental protective and risk factors (interaction across levels and functions in Fig. 1; see^14,44^). For example, although their exact contribution remains to be understood, some factors, such as reduced distinctiveness of auditory cortical speech representations, could represent a risk for developing dyslexia^45^, while other factors, such as strong verbal reasoning, vocabulary and attention skills, or a positive self-concept, seem to protect individuals from developing reading problems^46,47^. One challenge that applies here and was identified on several occasions during the workshop, is the difficulty of distinguishing these protective factors from compensatory processes that a child develops to circumvent already existing weaknesses in the brain’s developing reading network.

### How to capture developmental dynamics?

The examples above emphasize the complexity of child development, where levels of change as well as emerging functional networks continually interact in an idiosyncratic way. This raises the daunting question of how we can capture these developmental dynamics. What are the limitations we are currently confronted with and what are the potential solutions? During the interactive sessions (Open Space event, discussions, and working group sessions), different perspectives and expertise of all participants were integrated to jointly identifying the current main gaps in our knowledge and tools, namely (1) the need to build and use common frameworks, (2) using longitudinal, large-scale, multisite studies with representative participant samples, (3) understanding sources of interindividual variability, (4) explicit distinction of research aimed at understanding versus predicting, and (5) reproducible research.

#### Gap and solution 1: Common frameworks

We suggest that a common developmental framework that encompasses the multiple levels and functional networks and their dynamic interactions across time (Fig. 1) should be used across studies and disciplines (Fig. 1 is a suggestion for how such a framework could look like). Studies encompassing different levels and functions at multiple time points across development are currently scarce, but researchers who were present at the workshop all agreed that these are key to further advance our understanding of neurodevelopmental dynamics. Given that such studies involve multiple researchers from different sites and disciplines, a common framework is essential in order to allow communication between the experts at each level. It enables researchers to specify and communicate which levels and functional networks their study addresses and to take into account constraints and modulatory influences from other levels and functional networks. A common framework is also highly valuable for more exploratory research designed to generate hypotheses and to identify gaps in existing knowledge that can then be related to specific parts of the multilevel developmental model.

#### Gap and solution 2: From convenience samples to large-scale representative samples

A second issue is formed by the widely used convenience samples^48^, which often consist of a biased selection and relatively small number of participants. Researchers at the workshop emphasized that multisite studies are an important step to scale studies to large, representative samples, which is needed for analyses that allow to integrate multiple levels, functions, and time points. In the past decade, steps have been taken to merge datasets from multiple sites, and more recently, efforts have also been made to include multiple level data (e.g., ENIGMA dataset that provides large-scale brain and genetic data), and multiple time point data (e.g., ABCD dataset that provides longitudinal neuroimaging data during adolescence). In the future, these types of large-scale datasets should be further extended to include longitudinal data of early development, to cover even more levels and functions, and to include multicultural data^49^. Concerning the latter, given the strong impact of the context and environment during development, using more representative samples in terms of socioeconomic status and cultural background, and conducting multisite studies with worldwide coverage are essential. For such studies to be successful they must be based on a common framework (see “gap and solution 1”), make use of measures that are comparable across studies, and allow for reproducible analyses such as via pre-registered studies (see “Gap and solution 5”)^50,51^.

Although large-scale studies seem vital to further advance our research field, researchers at the workshop also argued that these need to be complemented by exploratory studies, which are generally conducted in smaller samples and can target specific parts of the multilevel/function developmental framework. Exploratory research aims at building a knowledge base and generation of novel hypotheses^52^. Especially for a relatively young field of research such as developmental cognitive neuroscience, exploratory studies are essential, and allow for subsequent theory construction. Ideally, these studies should be aware of the different levels of influence and be based on a common framework (see “Gap and solution 1”), to be able to advance such a framework with more evidence and detail. Hence, exploratory research and large-scale studies are complementary in that exploratory research can generate hypotheses and theories that can later be tested in large-scale studies that additionally allow testing the interactions with other levels, functions, and time points.

#### Gap and solution 3: Intra- and inter-individual variability

A third domain where much progress can be made, is in our understanding and analysis of interindividual variability^53,54^. Predominant analytical strategies treat this variability as noise and focus on central tendencies among groups of participants. In interpreting developmental data it is often essential to filter out random or task-irrelevant variability, e.g., related to measurement noise, different levels of stress, motivation, or mood states. This approach however also eliminates meaningful types of individual variability, reflecting for example intrinsic (genetic) factors or learning-induced variability involving different cognitive strategies or compensatory processes. Such interindividual variability is highly relevant in explaining the dynamic and idiosyncratic nature of functional brain development and is predicted by multidimensional continuous models of dysfunction. Thus, a central objective for future research that was identified during the workshop is the design of paradigms and data analysis strategies that enable us to utilize meaningful individual variability and distinguish it from variability due to noise.

At the brain level, functional MRI studies have traditionally used group average approaches (i.e., random-effects analyses which compare average activation maps per group), yet the average differences obtained via this approach do not necessarily reflect differences in activity but might just reflect that the activation is less/more consistent across individuals in a certain group. Therefore, in recent functional MRI studies^55,56^, group averaged analyses are complemented by measurements of interindividual consistency. This is done by creating penetrance maps which quantify the percentage of subjects that have significant activation in each voxel or in a predefined region of interest. A similar approach was proposed by Rosenblatt, et al.^57^. Another area of methods development where individual differences are taken into account is clustering (see for example^58^) and the use of group-specific brain templates to account for differences in brain structure when comparing children and adult groups^59^. Information on individual variability in the extent to which different brain regions are used to perform a certain task can ultimately provide us insight in potential compensatory mechanisms for children with atypical development.

To enable these types of analyses we need statistical methods that go beyond group averages and that harness the power of variability without producing spurious results, such as dynamic network models or latent change models. These models capture not only the average change over time (like standard approaches), but also the variability of this change and the extent to which this change is dependent on the score at the first time point^60^. Estimation of individual differences in learning-related change over time (individual growth rates) in addition to learning outcomes, can help identifying children at risk of developing e.g., math problems^61^. Another example where change scores can be informative is the observation that the dynamic coupling between reading and IQ over time discriminates typical readers from dyslexic readers^62^.

#### Gap and solution 4: explaining versus predicting developmental changes

A fourth issue that was identified is the importance of making clear whether a given study aims at explaining versus predicting developmental changes. Explanatory research on reading development for example, aims to understand how visual cortical regions specialize for letter recognition and how these letters start to be associated with speech sounds in the auditory cortex^26,28,29,63^. This research is important for understanding how learning to read changes the brain and why this forms an obstacle in struggling readers. However, it does not enable us to accurately predict individual differences in reading development, for which we need predictive research questions^64^. Both types of analysis serve their own important purpose: predicting outcomes can provide vital insights on the need for additional support, e.g., in terms of early intervention, or policy^65^, while methods that focus on understanding, can highlight underlying mechanisms that can be targeted by this intervention.

Standard analyses like regression optimize model fit, that is, they try to find the coefficients of a set of variables that best describes the dataset at hand. This leads to optimal understanding of the data as the coefficients resemble the best explanation of the data. Prediction approaches like machine learning do not focus on the best fit of the data at hand, but try to optimize out-of-sample prediction. That is, they try to choose coefficients in a way that minimizes the prediction error of new (out-of-sample) data. The coefficients are thus not optimized for explanation, but for prediction^66^. While both serve complementary goals towards increasing our understanding of developmental dynamics including individual variation^67^, highlighting the purpose of each study is important to integrate and interpret results in one common framework.

#### Gap and solution 5: Reproducible research

A fifth recurrent issue throughout the workshop was the need for reproducible research, with specific challenges for the field of developmental cognitive neuroscience^68^. In discussing “Gap and solution 2”, the importance of using large and representative samples to improve reproducibility in developmental cognitive neuroscience^49^ was already mentioned. In addition, replicability and transparency can be improved by open science initiatives such as making data and code available to other researchers^69^ and pre-registered studies, in which the research plan is written up before the actual implementation of the study. The extent of detail in the pre-registered reports vary greatly and often depend on the platform used (e.g., OSF, Center for Open Science), but the basic idea is that the set-up and the analyses plan is already determined before data collection and post hoc adjustments should be explicitly stated. Another option is Registered Reports, which is a type of research article for which the peer review is mainly conducted before data collection and based on the background literature, hypotheses, and methods including the planned analysis, hence publication of the work does not depend on the obtained results^51^. Developmental journals are increasingly enabling this type of articles. For purely exploratory research that is not hypothesis-driven, this is not the preferred format, but for other types of studies this is a promising approach that allows unbiased publication of results. Researchers at the workshop expected the use of pre-registered studies and registered reports to further rise in the future.

## Conclusions

To conclude, several directions for future research ensued from the workshop. First, to capture the complexity and variability of developmental brain dynamics we need a common framework across levels, functions, and time. This will enhance collaboration and unify the research on multidimensional developmental dynamics, as it enables researchers to indicate which part(s) of the framework their work addresses, while also increasing awareness of other influences. Second, exploratory and hypothesis-driven research are both important, and which of the two is the (main) purpose of a study should be transparent. Similarly, we need studies aimed at explaining developmental dynamics, as well as studies aimed at predicting certain developmental outcomes. Again, it is important that these aims are clearly distinguished by researchers. Third, large-scale longitudinal cross-center developmental studies are needed that use new advances in methods, hardware, and open science. Such endeavors are needed to distinguish relevant individual differences, to include multiple levels and time points, to increase reliability and reproducibility, and to better integrate intercultural and global perspectives. While such large-scale studies typically involve hypothesis-driven research, especially in a relatively new field such as developmental cognitive neuroscience, exploratory studies with relatively lower samples are also needed. Finally, to stimulate collaboration among experts across a wide range of disciplines and backgrounds we need effective platforms. During the workshop, the idea of a “Scientific Tinder” arose, in which research(ers) can be matched on topic, data type, or analysis. Several of such initiatives already exist, for example the OSF-based “StudySwap”: https://osf.io/meetings/studyswap/. We highly encourage researchers to reach out to other groups beyond their own discipline, expertise, and cultural focus, to realize the research directions summarized above.
